# Severe Refractory Paraneoplastic Mucous Membrane Pemphigoid Successfully Treated With Rituximab

**DOI:** 10.3389/fmed.2019.00008

**Published:** 2019-01-29

**Authors:** Marcel Wittenberg, Margitta Worm

**Affiliations:** Department of Dermatology, Venerology and Allergology, Charité- Universitätsmedizin Berlin, Berlin, Germany

**Keywords:** mucous membrane pemphigoid, rituxmab, paraneoplastic, laminin 332, therapy refractoriness

## Abstract

Mucous membrane pemphigoid (MMP) is a rare autoimmune bullous disease of the mucous membranes, which can cause irreversible scarring and is discussed to be associated with cancer, if laminin-332-autoantibodies are present. MMP with severe ocular and laryngeal involvement is difficult to treat and can be treatment-refractory to conventional immunosuppressant therapy. A 67-year-old man with a history of prostate cancer presented to our clinic with sore throat, intraoral bullae, odynophagia, dysphonia, exertional dyspnea, and erosions of the glans penis. Clinical examination confirmed a laryngo-pharyngitis with involvement of the epiglottis and bilateral symblepharon. Diagnostics comprising multiple biopsies, direct and indirect immunofluorescence, serology analysis, and immunoblotting confirmed the diagnosis of a paraneoplastic MMP by showing a subepithelial split in histology and the presence of anti-laminin-332-antibodies. Despite combined systemic treatment with prednisolone and either dapsone or azathioprine, a progress of the disease occurred leading to severe ocular and laryngeal complications. Two month after rituximab treatment, complete disease control was achieved. This case report shows a severe ocular and life threatening laryngeal involvement of therapy-refractory paraneoplastic MMP highlighting the importance of interdisciplinary management and difficulty of diagnosing MMP despite repeated diagnostic testing.

## Background

Mucous membrane pemphigoid (MMP) is defined as a heterogeneous group of autoimmune, chronic inflammatory blistering diseases, which lead to subepithelial bullae predominantly of the mucous membranes and occasionally the skin (1–3). The most common affected sites are the oral and ocular mucosae, but an involvement of the nasopharynx, esophagus, larynx, and anogenital region may also occur. The underlying pathophysiology is characterized by a linear deposition of IgG, IgA, or C3 along the epithelial basement membrane zone (1). If MMP is suspected clinically, diagnostic testing and treatment is required without delay in order to prevent complications like irreversible scarring potentially leading to blindness, airway stenosis, esophageal, and anogenital stricture (3). Smaller studies and case reports suggest positive laminin-332 (laminin-5)-autoantibodies to be associated with a paraneoplastic manifestation of MMP (4–7).

Epidemiological studies of MMP are rare. Thus, the real world incidence of MMP remains unknown. In the literature the incidence in United Kingdom of cicatricial conjunctivitis was calculated as 0.8 per million, whereas the incidence of MMP in France and Germany was estimated to be 1.3–2.0 per million per year (8–10).

Therapy of MMP is mainly dependent on the classification of high and low risk disease. Low risk MMP (involvement of oral mucosae and skin) should be treated initially by topical steroids whereas it is recommended to treat high risk MMP (involvement of the eyes, esophagus, larynx, urogenital region) by systemic corticosteroids. In case of incomplete disease control, dapsone in combination with immunosuppressive therapies like azathioprine, cyclophosphamide, or mycophenolate mofetil should be applied (1). According to the European guideline for management and treatment of bullous pemphigoid, rituximab is recommended as third-line therapy, if conventional immunosuppressive drugs were not effective, contraindicated, or showed unacceptable side effects (11). In the literature, rituximab has been described effective as treatment in therapy-recalcitrant MMP (12–16). However, relapse is frequent and only a few studies including a small quantity of patients are available (12–16).

Herewith we present a case of a MMP with a positive history of cancer, severe laryngeal, ocular, and genital involvement showing a refractory course of the disease on azathioprine and dapsone immunosuppressive treatment. Given the severe involvement of the eyes and epiglottis we emphasize the indispensable multidisciplinary management of paraneoplastic MMP.

## Case Presentation

A 67-year-old caucasian male patient presented first to the Clinic for Dermatology in August 2017 suffering since March 2017 from sore throat, intraoral bullae, odynophagia, dysphonia, exertional dyspnea, and erosions of the glans penis. He was first treated by his general practitioner for a suspected oral herpes infection with antiviral medication without improvement. At the onset of the symptoms the patient had been retired.

The medical history of the patient revealed a history of prostate cancer diagnosed and treated by radical prostatectomy ~1 year before the onset of symptoms, epilepsy treated with levetiracetam since 2002, asthma and a chronic rhinosinusitis since 1988 treated with surgery.

**Figure 1:**
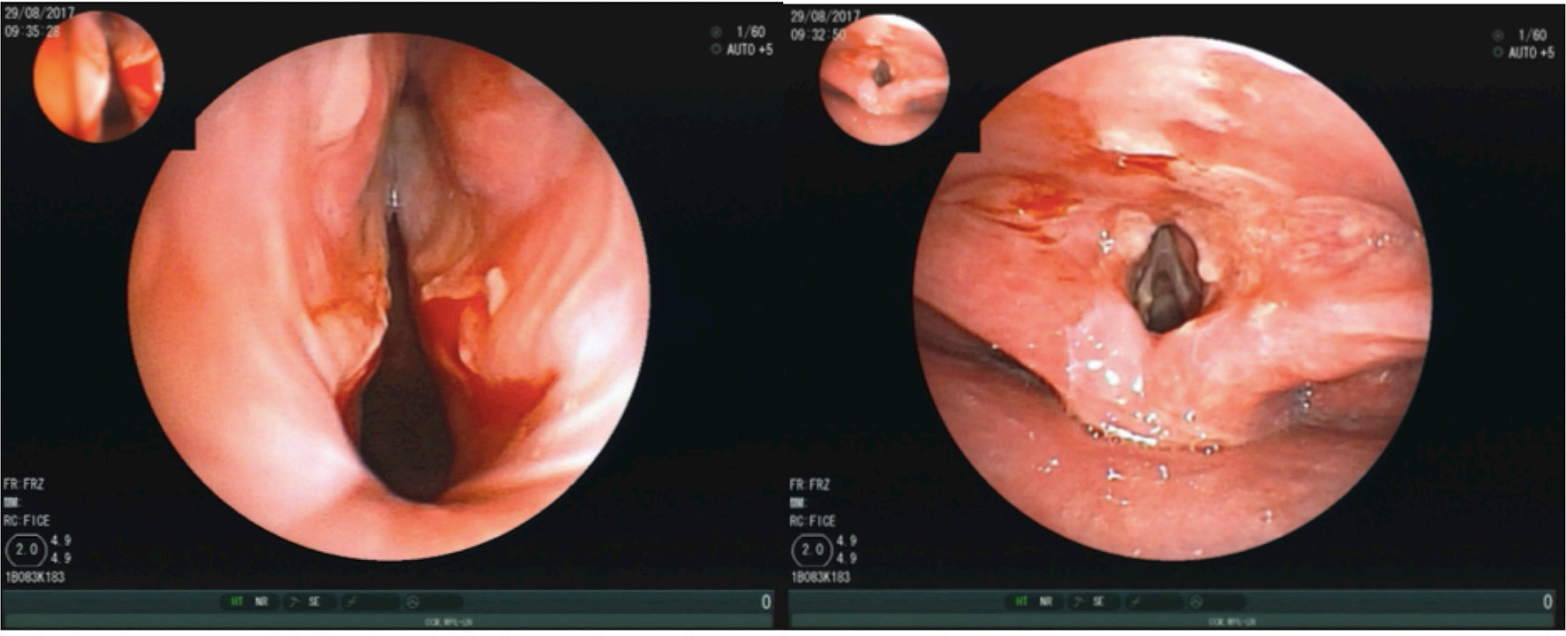
Bronchoscopy showing multiple ulcers of the pharynx, highly vulnerable mucous membranes and granulomatous changes of the vocal cords.

The clinical examination revealed dry mucuous membranes in the oral cavity with erosions and swellings of the buccal mucosa and the hard palate. Inspection of the pharynx showed a distinct laryngo-pharyngitis with involvement of the epiglottis. To exclude an involvement of trachea a bronchoscopy was done revealing multiple ulcers of the pharynx, highly vulnerable mucous membranes and granulomatous changes of the vocal cords (Figure 1).

**Figure 2:**
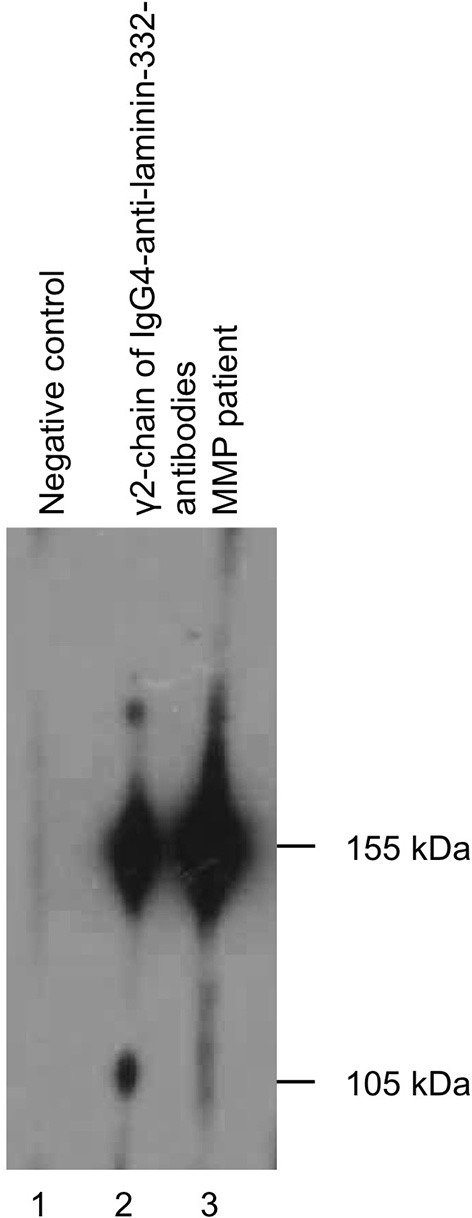
Immunoblotting of extracellular matrix showing circulating IgG4-autoantibodies to γ2-chain of laminin-332.

A biopsy, taken shortly before the first presentation to our clinic in an external hospital showed a subepithelial split together with an inflammatory cell infiltration comprising monocytes and granulocytes. The DIF analysis was negative. In our clinic an additional biopsy of the oral mucous membrane stained with haematoxylin and eosin staining was done. The result was negative for MMP showing an increase of collagen fibers with lymphohistiocytic infiltrate and an increased amount of plasma cells in the corium. The DIF analysis revealed unspecific perivascular C3 deposits. Consistent with the first biopsy, a third biopsy with haematoxylin and eosin staining, showed a subepithelial split (Table 1). Indirect immunofluorescence using both monkey esophagus and human salt-split skin did not detect circulating IgG- or IgA-autoantibodies. In addition, serum analysis using ELISA with recombinant BP180 NC16A, BP180, BP230, and desmoglein 1 and 3 was negative (Table 2). As serology testings were negative, immunoblotting of extracellular matrix was performed, which was positive for circulating IgG4-autoantibodies to γ2-chain of laminin-332 (Figure 2). The differential diagnosis of Behçet's disease presenting orogenital ulceration was unlikely as the patient only fulfilled one minor criteria, did not show characteristic histological changes for Behçet's disease or any other major or minor criteria for Behçet's disease. Accordingly, clinical criteria such as uveitis or retinal vasculitis, characteristic skin lesions, HLA-typing for B51 and pathergy test were negative. The differential diagnosis of a cytotoxic-mediated disease like Stevens-Johnson-Syndrome was rather unlikely, given the course of the disease, the affected sites, the lack of a possible trigger and the histological findings without signs of a CD8+-mediated reaction like an interface dermatitis or necrotic keratinocytes. An oral candida infection was excluded by a swap. Given the positive history for prostate cancer we performed a tumor staging. The chest-x-ray, ultrasound of the abdomen and PSA-value (0.1 μg/l) were within normal limits. Based on the clinical course, the histological finding and the immunoblot positive for laminin-332-autoantibodies, we suspected a paraneoplastic MMP.

**Table 1:** Histological findings of performed biopsies.

**Biopsy No**.	**Hematoxylin and eosin staining**	**DIF analysis**
1	Subepithelial split together with an inflammatory cell infiltration comprising monocytes and granulocytes	Negative
2	Increase of collagen fibers with lymphohistiocytic infiltrate and an increased amount of plasma cells in the corium	Negative
3	Subepithelial split	Not done

**Table 2:** Laboratory findings.

**Method**	**Substrate analyzed**	**Positive or negative result**
ELISA	Recombinant proteins: BP180 NC16A, BP180, BP230, desmoglein 1, desmoglein 3	Negative
Immunoblot	Extracellular matrix	Positive for γ2-chain of laminin-332-autoantibodies
Indirect immunofluorescence	Monkey esophagus and human salt-split skin	Negative

Due to an acute exacerbation with progressive exertional dyspnea, anxiety choking, dry cough, hoarseness and ocular irritation a chest-x-ray, and body plethysmography were performed to exclude an acute exacerbation of asthma. Because of exertional dyspnea a laryngoscopy was performed which revealed progressive oral ulcers as well as a synechia of the first third of the vocal cords.

**Figure 3:**
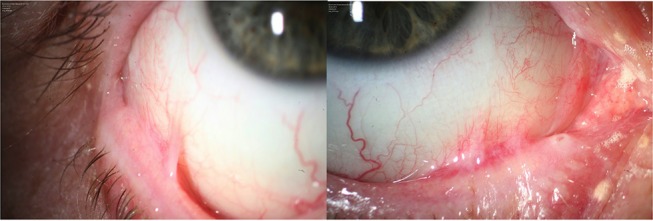
Active bilateral stage 4 symblepharon according to Tauber und Foster classification.

Even though the diagnosis could not be confirmed by immunohistological criteria at the time of the first symptoms, a paraneoplastic MMP was suspected based on the clinical manifestation with the positive cancer history. Given both, the critical laryngal involvement causing dyspnoea and the ocular bilateral stage 4 symblepharon according to Tauber und Foster classification (17) (Figure 3), systemic treatment was initiated. Intravenous methylprednisolone was applied (250 mg/day) at 3 consecutive days. The pulse therapy was repeated for three times every 4 weeks. Oral therapy with dapsone (100 mg/day), which had been initiated after the first pulse therapy was discontinued by the general practitioner due to methemoglobinemia, cyanosis of the lips, and dyspnoea. Instead a combined oral therapy comprising azathioprine (100 mg/day) and prednisolone (50 mg/day) was given. Prednisolone was consecutively reduced to 10 mg per day. Topical treatment included Hylogel due to ocular involvement, inhalation of Tacholiquin 1% and a prednisolone-dexpanthenol solution. Hereafter disease control was achieved with reduction of hoarsness and dyspnoea. Azathioprine was discontinued after 4 month due to elevated values of gamma-glutamyltransferase.

Due to an acute laryngotracheitis with acute dyspnea as well as inspiratory and expiratory stridor, a microlaryngoscopy with division of the synechia of the anterior commissure was performed in the clinic for ear, nose, and throat followed by a fixation of a silicone sheet.

**Figure 4:**
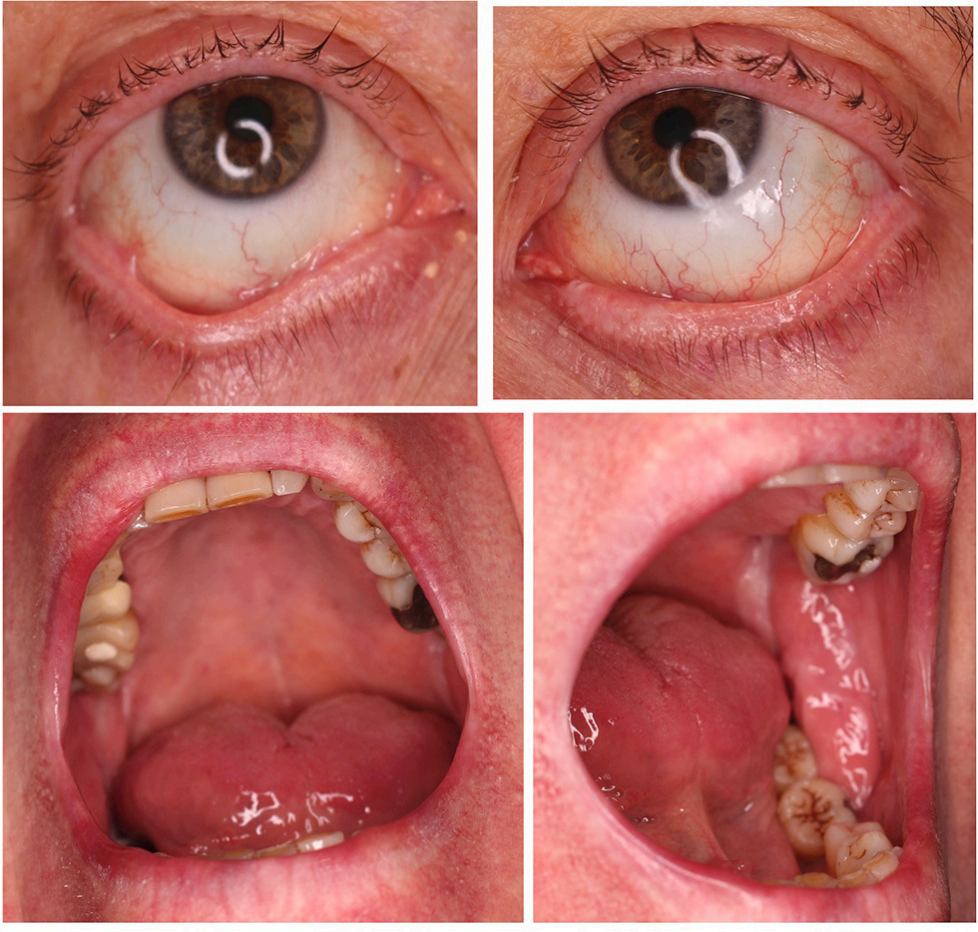
Post-treatment pictures showing a stable ocular involvement and no new enoral lesions 5 month after rituximab treatment.

Given both systemic treatments with azathioprine and dapsone had to be discontinued due to adverse effects, therapy with rituximab 1,000 mg was initiated twice in a 14-days interval. The follow-up examination 8 weeks later revealed a stable disease with no new oral lesions (Figure 4). According to the patient dyspnea did not appear since the start of rituximab treatment. The ocular manifestation of the MMP was assessed stable by the ophthalmologists. During the latest check-up for cancer no signs of relapse were detected. Differential white blood cell count was taken during and after the treatment with rituximab. Initially, total leukocytes and lymphocytes were within normal limits (Leukocytes: 6.75/nl, lymphocytes: 1.13/nl). 7 weeks after the second treatment with rituximab a lymphocytopenia was detected (0.60/nl). Leukocytes and lymphocytes before and after radical prostatectomy were normal (leukocytes: before 6.44/nl, after 9.90/nl, lymphocytes: before 1.12/nl, after 1.24/nl)

## Discussion

MMP is a rare autoimmune blistering disease, which predominantly affects the mucous membranes and is characterized by the linear deposition of IgG, IgA, or C3 along the epithelial basement membrane zone (1). The diagnosis of MMP is mainly based on clinical manifestation, immunohistological examination as well as histopathological and serology analysis like indirect immunofluorescence, immunoblotting, or immunoprecipitation techniques (3). According to the consensus conference the clinical course of disease as well as the DIF analysis are crucial criteria to diagnose a MMP (1).

Diagnosing MMP can be challenging (18). The diagnosis of MMP in our patient at the time of the first hospitalization was made by clinical features only, as the first diagnostic workup including histology, serology, and immunohistology remained negative. As the DIF of the biopsy taken in an external hospital did not show specific changes for MMP, an additional biopsy was taken from the buccal mucosa. Again, the histology and DIF analysis did not show characteristic changes for MMP. According to the literature false-negative results at the first diagnostic workup of MMP are not rare (18). Repeated testing is highly recommended to increase the sensitivity of the DIF analysis for MMP diagnosis (18). However, DIF analysis can show false-negative results especially in patients with only ocular mucous membrane involvement or when longstanding lesions are being analyzed (3, 19). A third biopsy taken of the laryngeal mucosa showed a subepithelial split in hematoxylin and eosin staining consistent with the diagnosis of a MMP. Unfortunately an additional DIF analysis of the third biopsy has not been arranged by the ENT department. Shimanovich et al. state, that despite repeated testing up to 5% of the biopsies taken from patients with MMP remain negative (18). Consistent with our report, the diagnosis of MMP could be confirmed by the detection of circulating autoantibodies against bullous pemphigoid antigen 180 or laminin-332 (18).

For ocular MMP Labowsky et al. have shown that patients showing a linear deposition of IgG, IgA, or C3 at the basement membrane in a DIF biopsy were more likely to be treated with systemic immunosuppression compared to patients showing no linear immunologic deposits at the basement membrane zone in a DIF biopsy (19). They conclude that even without confirming the diagnosis by DIF, treatment with systemic immunosuppression should be initiated (19). In our case systemic treatment was initiated due to the reduced general condition of the patient as well as the risk of irreversible complications due to a delay of intervention. Due to progressive disease with severe dyspnea and the need of discontinuation of systemic dapsone and azathioprine therapy, rituximab treatment was initiated. In the literature, large randomized controlled studies analyzing rituximab treatment in recalcitrant MMP are sparse. Lamberts et al. investigated the effectiveness and safety of rituximab in 28 patients with recalcitrant pemphigoid diseases (13). Disease control was achieved in the majority of the cases (67.9%). However, during follow-up, 66.7% patients relapsed. Repeated treatment with rituximab was effective in 85.7% of retreated cases. Interestingly, MMP patients showed the most benefit of rituximab (disease control in 85.7%) compared to other pemphigoid diseases. However, the rate of relapses was high (75%). The best outcome was achieved using the high dose protocol (1,000 mg rituximab at days 1 and 15) compared to the low dose protocol (500 mg rituximab) (13). In comparison to IgG-dominant pemphigoid diseases, rituximab was less effective in IgA-dominant pemphigoid diseases, representing an unresponsiveness of IgA positive plasma cells to rituximab (13). During the follow-up, 3 patients died of which one was probably treatment related. Rübsam et al. analyzed 6 MMP patients with ocular involvement after being treated with rituximab (14). Using the high dose protocol all patients responded to rituximab. However, relapse occurred in 83.3% of the cases. Consistent with the results of Lamberts et al. repeated treatment with rituximab lead to remission in all patients (14). Two patients died probably not related to the treatment (14). Heelan et al. investigated 8 patients with MMP being treated with 1,000 mg rituximab at days 1 and 15 (12). After disease control was achieved in all patients, a relapse occurred in 100% of the cases. Retreatment with rituximab lead to a 100% response rate (12). Shetty et al. published a retrospective study including case series and case reports of 28 patients with MMP (15). In this study, different protocols of rituximab treatment were applied. Consistent with the previous studies a high response and relapse rate was described (disease control 82.1%, relapse in >50%) (15). Tomsitz et al. investigated a cohort of 22 patients with recalcitrant autoimmune blistering diseases using the high dose rituximab protocol (16). Seventy-two percent of the patients showed a partial or complete remission after the first cycle. However, unlike the findings of Lamberts et al. the response rate in patients with MMP was low (40%) (16). In our case, disease control was achieved 2 month after administering rituximab using a high dose protocol. Since 5 months after the treatment no relapse of MMP or adverse effects have been reported.

Adjuvant treatment with intravenous immunoglobulin or immunoadsorption in combination with rituximab has been described effective to treat therapy-refractory MMP (5, 20–22). Protein A immunoadsorption describes a method to selectively remove circulating antibodies from the blood in an extracorporeal circuit (23). It is recommended to be used as first line treatment in pemphigus vulgaris, pemphigus foeliaceus, paraneoplastic pemphigus vulgaris, or epidermiolysis bullosa aquisita (24). Albeit complete disease control was achieved in our case by rituximab monotherapy, relapse of the disease after rituximab treatment is frequent in ocular MMP (12). Thus, protein A immunoadsorption or immunoglobulin treatment represent a promising alternative to rituximab in case of recurrence.

The patient presented here had circulating IgG4-autoantibodies to the γ 2-chain of laminin-332-autoantibodies determined by immunoblotting analysis. Even though studies and case reports suggest an association between detection of laminin-332-autoantibodies and paraneoplastic MMP, Bernard et al. could not detect a significant relationship in a multicenter retrospective study (4, 6, 7, 25). The patient's history was positive for a prostate cancer diagnosed and treated ~1 year before the onset of the first symptoms. A staging examination remained negative, therefore a direct link between progressive oncological disease and MMP has not been verified in our case. Further investigations regarding the interaction of neoplasms and anti-laminin-332-antibodies are required.

Multidisciplinary management and treatment of the MMP is of the utmost importance, as different sites can be affected (1). Unlike the majority of the cases with involvement of the oral mucosae and the eyes, the patient presented with severe involvement of the larynx and epiglottis leading to an acute laryngo-pharyngitis, synechia of the vocal cords and recurrent episodes of severe dyspnea. On account of the close cooperation with the clinic for ear, nose, and throat, the division of the synechia was performed, leading to an improvement of the symptoms. Herewith we emphasize that the optimal outcome for the patient was achieved only due to multidisciplinary management including ENT specialists and ophthalmologists. A multidisciplinary follow-up is highly recommended to ensure best disease management.

For the publication of this case report written informed consent from the patient in accordance with the Declaration of Helsinki was obtained.

## Author Contributions

MW and MD writing of the manuscript, literature research, figures. MW revision of the manuscript, initiation of the publication.

### Conflict of Interest Statement

The authors declare that the research was conducted in the absence of any commercial or financial relationships that could be construed as a potential conflict of interest.
